# BK Polyomavirus Hijacks Extracellular Vesicles for *En Bloc* Transmission

**DOI:** 10.1128/JVI.01834-19

**Published:** 2020-02-28

**Authors:** Lynda Handala, Emmanuelle Blanchard, Pierre-Ivan Raynal, Philippe Roingeard, Virginie Morel, Véronique Descamps, Sandrine Castelain, Catherine Francois, Gilles Duverlie, Etienne Brochot, Francois Helle

**Affiliations:** a EA4294, Agents Infectieux, Résistance et chimiothérapie (AGIR), Centre Universitaire de Recherche en Santé, Université de Picardie Jules Verne, Amiens, France; b Laboratoire de Virologie, Centre Hospitalier Universitaire, Amiens, France; c Plateforme IBiSA de Microscopie Electronique, Université de Tours et CHU de Tours, Tours, France; d INSERM U1259, Université de Tours et CHU de Tours, Tours, France; International Centre for Genetic Engineering and Biotechnology

**Keywords:** BKPyV, polyomavirus, extracellular vesicles, neutralizing antibodies, quasienveloped virus, *en bloc* transmission, JCPyV, MCPyV, TSPyV, SV40

## Abstract

Most people are asymptomatic carriers of the BK polyomavirus (BKPyV), but the mechanisms of persistence and immune evasion remain poorly understood. Furthermore, BKPyV is responsible for nephropathies in kidney transplant recipients. Unfortunately, the sole therapeutic option is to modulate immunosuppression, which increases the risk of transplant rejection. Using iodixanol density gradients, we observed that Vero and renal proximal tubular epithelial infected cells release two populations of infectious particles, one of which cosediments with extracellular vesicles (EVs). Electron microscopy confirmed that a single vesicle could traffic tens of viral particles. In contrast to naked virions, the EV-associated particles (eBKPyVs) were not able to agglutinate red blood cells and did not use cell surface sialylated glycans as an attachment factor, demonstrating that different entry pathways were involved for each type of infectious particle. However, we also observed that naked BKPyV and eBKPyV were equally sensitive to neutralization by the serum of a seropositive patient or commercially available polyvalent immunoglobulin preparations, which occurred at a postattachment step, after endocytosis. In conclusion, our work shows a new mechanism that likely plays a critical role during the primary infection and in the persistence, but also the reactivation, of BKPyV.

**IMPORTANCE** Reactivation of BKPyV is responsible for nephropathies in kidney transplant recipients, which frequently lead to graft loss. The mechanisms of persistence and immune evasion used by this virus remain poorly understood, and a therapeutic option for transplant patients is still lacking. Here, we show that BKPyV can be released into EVs, enabling viral particles to infect cells using an alternative entry pathway. This provides a new view of BKPyV pathogenesis. Even though we did not find any decreased sensitivity to neutralizing antibodies when comparing EV-associated particles and naked virions, our study also raises important questions about developing prevention strategies based on the induction or administration of neutralizing antibodies. Deciphering this new release pathway could enable the identification of therapeutic targets to prevent BKPyV nephropathies. It could also lead to a better understanding of the pathophysiology of other polyomaviruses that are associated with human diseases.

## Abstract (precis)

Reactivation of BKPyV is responsible for nephropathies in kidney transplant recipients, which frequently lead to graft loss. The mechanisms of persistence and immune evasion used by this virus remain poorly understood, and a therapeutic option for transplant patients is still lacking. Here, we show that BKPyV can be released into EVs, enabling viral particles to infect cells using an alternative entry pathway. This provides a new view of BKPyV pathogenesis. Even though we did not find any decreased sensitivity to neutralizing antibodies when comparing EV-associated particles and naked virions, our study also raises important questions about developing prevention strategies based on the induction or administration of neutralizing antibodies. Deciphering this new release pathway could enable the identification of therapeutic targets to prevent BKPyV nephropathies. It could also lead to a better understanding of the pathophysiology of other polyomaviruses that are associated with human diseases.

## INTRODUCTION

Most people are asymptomatic carriers of the BK polyomavirus (BKPyV). After acquisition in early childhood, the virus establishes persistent infection in the kidney and urogenital tract epithelial cells, but the mechanisms of persistence and immune evasion remain poorly understood. BKPyV can also be reactivated and induce various complications in some patients, especially in cases of immunosuppression. Reactivation of BKPyV is thus responsible for hemorrhagic cystitis in up to 15% of bone marrow transplant recipients and for nephropathies (BK virus nephropathy [BKVN]) in up to 10% of kidney transplant recipients, which frequently lead to graft loss (1). Currently, the only therapeutic option for kidney transplant patients is to modulate immunosuppressive treatment in order to control infection, but this increases the risk of transplant rejection. Recent studies have suggested that patients with high titers of neutralizing antibodies to the replicating strain had a lower risk of developing BKPyV viremia and that prevaccination against all serotypes might offer protection against graft loss or dysfunction due to BKVN (2, 3). However, such a vaccine is still lacking.

A better understanding of the BKPyV life cycle could permit identification of new therapeutic targets to inhibit virus replication (4). In particular, only a few studies have been dedicated to understanding the mechanisms of virion assembly and release. After translation, the VP1, VP2, and VP3 capsid proteins are translocated into the nucleus to assemble with viral genomes and form progeny virions (5). Then, naked virions are expected to be released after cell lysis. However, lytic infection is questionable *in vivo*, since the virus establishes infection that persists for life in healthy immunocompetent carriers. Furthermore, Evans et al. recently provided evidence for a nonlytic release pathway of BKPyV virions (6).

Here, we show that BKPyV can be released within extracellular vesicles (EVs). We call these virus-containing vesicles enveloped BKPyVs (eBKPyVs). We also demonstrate that these eBKPyVs do not interact with cell surface sialylated glycans and compare their sensitivity to neutralizing antibodies with that of naked particles. This mechanism likely plays a major role in viral persistence.

## RESULTS

### BKPyV particles are released within EVs.

**FIG 1:**
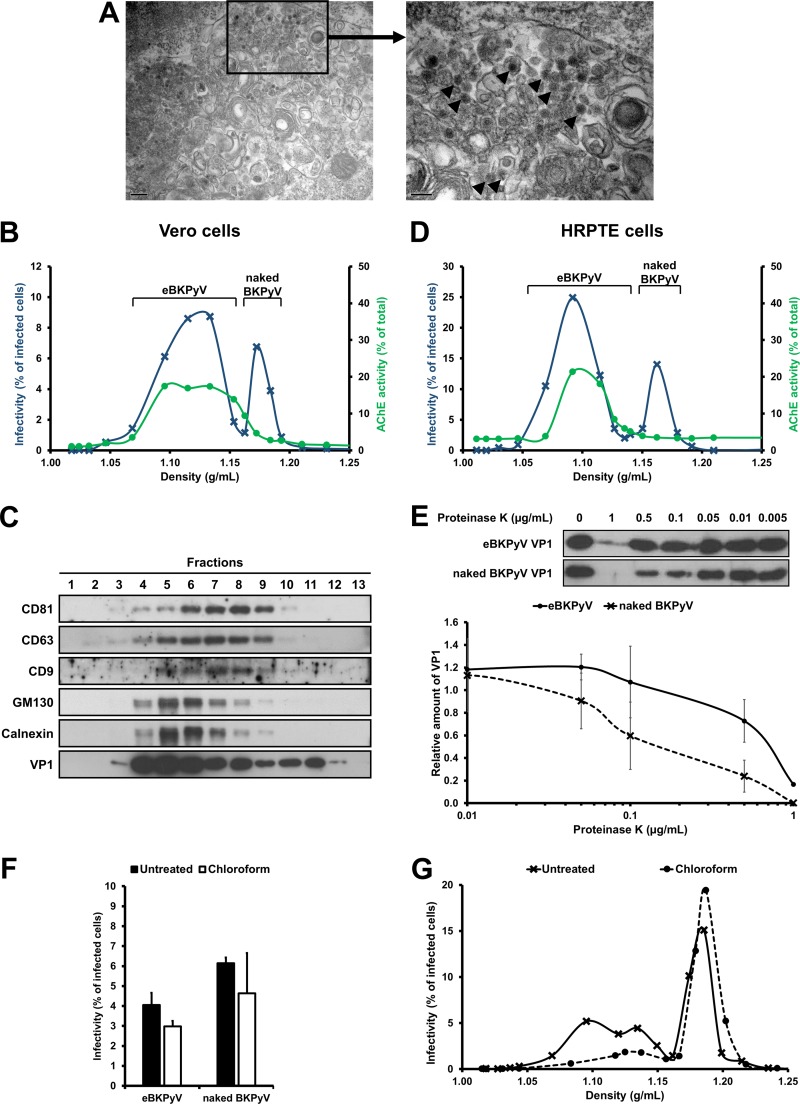
BKPyV particles are released within EVs. (A) Chronically infected Vero cells were fixed and processed for electron microscopy. Electron micrographs show the presence of viral particles (indicated by arrowheads) in MVBs. The right panel (bar, 100 nm) corresponds to an enlargement of the left panel (bar, 0.2 μm). (B) Vero cells were infected with BKPyV at an MOI of 1. Supernatant was harvested 3 days postinfection, filtered at 0.45 μm, and overlaid on a 20% to 45% (wt/vol) iodixanol gradient. After a 24-h ultracentrifugation, 17 fractions were collected. The density (g/ml) of each fraction was calculated according to the optical density at 340 nm. BKPyV infectivity in each fraction was assessed by immunofluorescence 3 days after infection of naive Vero cells. It is expressed as percentages of infected cells. AChE activity was analyzed to detect the presence of EVs in each fraction. (C) EVs contained in the supernatant of infected cells were concentrated 100× by PEG precipitation and overlaid on a 20% to 45% (wt/vol) iodixanol gradient. The presence of EVs in fractions 1 to 13 was evaluated by the detection of CD9, CD63, and CD81 by Western blotting. The presence of viral capsids was evaluated by the detection of VP1. The presence of contaminating small cellular organelles was evaluated by the detection of GM130 and calnexin. (D) The experiment was performed as for panel B with the supernatant of HRPTE cells, harvested 10 days postinfection. (E) Fractions containing eBKPyV (fraction 6) or naked BKPyV (fraction 12) were treated with different concentrations of proteinase K for 10 min. The volumes of the fractions were adjusted to treat similar amounts of the VP1 protein under both conditions. The sensitivity to proteinase K digestion was then assessed by detection of the VP1 capsid protein by Western blotting (top panel) and evaluated by quantifying the relative amount of VP1 on the Western blots using ImageJ (bottom panel). Results are reported as the means ± standard deviations from two independent experiments. (F) After iodixanol gradient ultracentrifugation, fractions containing eBKPyV or naked BKPyV were treated with chloroform or left untreated and then analyzed for infectivity on naive Vero cells. (G) The supernatant of Vero cells was harvested 4 days postinfection and treated with chloroform or left untreated before performing the buoyant density iodixanol gradient ultracentrifugation. Results presented in panels B, D, F, and G are means from duplicates from representative experiments.

Evans et al. recently showed that endosomes were involved in a nonlytic BKPyV release pathway (6). On our side, by performing electron microscopy on chronically infected Vero cells, we observed the presence of viral particles in the multivesicular bodies (MVBs), a specialized subset of endosomes (Fig. 1A). Since MVBs can fuse with the plasma membrane to release exosomes, we hypothesized that the nonlytic BKPyV release pathway could involve EVs. We thus decided to characterize the infectious particles released by infected Vero cells. Using iodixanol gradients, we observed the existence of two populations of BKPyV infectious particles (Fig. 1B). The population with the higher density peaked at 1.18 g/ml and likely corresponded to naked virions, consistent with the densities reported for the related JC polyomavirus (JCPyV) and Merkel cell polyomavirus (MCPyV) in such gradients (7, 8). In contrast, the second population of infectious particles, called eBKPyVs, exhibited a density ranging between 1.05 and 1.15 g/ml, which was consistent with membrane association. We confirmed that this population cosedimented with EVs by assessing the acetylcholinesterase (AChE) activity (Fig. 1B) as well as the presence of the tetraspanin membrane proteins CD9, CD63, and CD81, known to be enriched in these vesicles (Fig. 1C). We also considered the cosedimentation with contaminating small cellular organelles by assessing the presence of GM130 and calnexin. However, we observed that these contaminants peaked in fractions 5 and 6, in contrast to EVs and infectious eBKPyVs, which peaked in fractions 7 and 8 (Fig. 1C). Importantly, we easily detected the presence of the VP1 capsid protein not only in the naked BKPyV fractions but also in the eBKPyV fractions, strongly suggesting the presence of full viral particles in both types of fractions (Fig. 1C). We also demonstrated that this phenomenon was not cell type specific, since similar results were obtained when working with primary human renal proximal tubule epithelial (HRPTE) cells, which are a more physiologically relevant model to study BKPyV infection (Fig. 1D).

As shown in Fig. 1E, when performing a proteinase K protection assay, which is commonly used to study the envelopment of viruses, we observed that the eBKPyV VP1 was less sensitive to proteinase K digestion than the naked BKPyV VP1 (Fig. 1E), suggesting that infectious particles of the eBKPyV fraction were within vesicles. We also investigated the effect of chloroform extraction, which is a classical method to distinguish enveloped from nonenveloped viruses. As shown in Fig. 1F, the treatment of eBKPyVs with chloroform had only a slight effect on their infectivity, excluding the possibility that naked BKPyV genomic DNA was transmitted to naive cells through EVs and confirming that infectious virions were present in this fraction. Furthermore, when the chloroform treatment was performed on the infected cell supernatants prior to the iodixanol gradient, we observed the disappearance of the eBKPyV population and a slight increase of the naked BKPyV population (Fig. 1G), consistent with enveloped particles rendered naked by chloroform treatment prior to the gradient. Thus, our results strongly suggested that infectious viral particles were contained within EVs.

**FIG 2:**
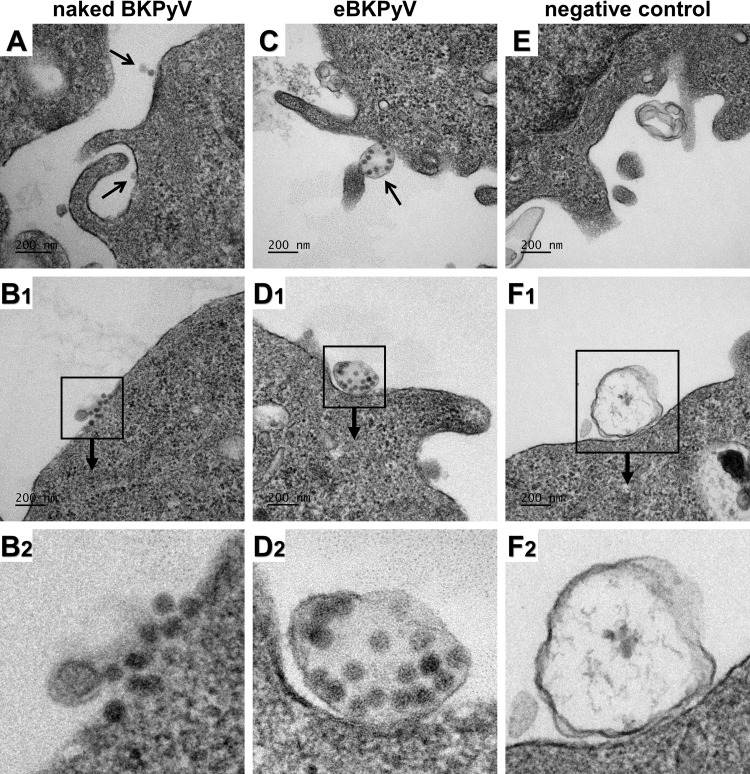
eBKPyVs correspond to EVs enclosing tens of viral particles. Vero cells were incubated for 2 h with fractions containing eBKPyV or naked BKPyV, fixed, and processed for electron microscopy. (A and B) Electron micrographs of naked particles at the surface of Vero cells. (C and D) Electron micrographs of eBKPyVs at the surface of Vero cells. (E and F) Electron micrographs of EVs free of virions at the surface of Vero cells. Naked particles and eBKPyVs are indicated by arrows in panels A and C, respectively. Panels B2, D2, and F2 correspond to enlargements of panels B1, D1, and F1, respectively.

To firmly confirm that eBKPyVs were contained within EVs and not attached to them, we performed electron microscopy. To get a sufficient amount of viral particles in the different fractions, we carried out a polyethylene glycol (PEG) precipitation before the iodixanol gradient. Vero cells were then incubated with eBKPyV- or naked BKPyV-enriched fractions, fixed, and processed for electron microscopy. As expected, isolated or grouped naked particles were clearly observed at the surface of Vero cells incubated with naked BKPyV-enriched fractions (Fig. 2A and B). In contrast, vesicles carrying one or more tens of viral particles were almost exclusively observed when cells were incubated with eBKPyV-enriched fractions (Fig. 2C and D). Vesicles that were similar but free of virions were observed with the corresponding fractions obtained from the supernatant of noninfected cells (Fig. 2E and F).

### eBKPyVs do not interact with gangliosides at the cell surface.

**FIG 3:**
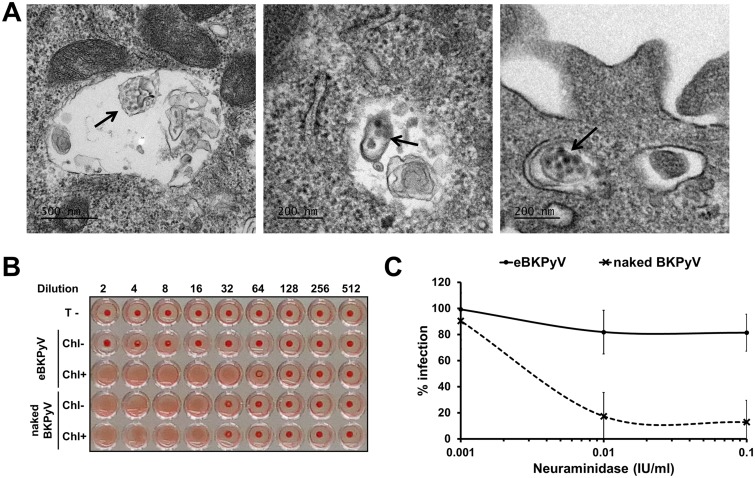
eBKPyVs do not interact with cell surface sialylated glycans. (A) Vero cells were incubated for 2 h with fractions containing eBKPyV, fixed, and processed for electron microscopy. Electron micrographs show intact EVs containing viral particles in endosomal compartments (indicated by arrows). (B) After iodixanol gradient ultracentrifugation, fractions containing eBKPyV or naked BKPyV were treated with chloroform (Chl+) or left untreated (Chl-). Then, serial dilutions of treated and untreated fractions were mixed with human RBCs from a type O Rh^+^ blood donor and allowed to settle in round-bottom wells overnight at 4°C. PBS was used as a negative control for hemagglutination (T-). Results of a representative experiment are shown. (C) Naive Vero cells were treated with neuraminidase for 1 h at 37°C, at the indicated concentrations. Then, cells were washed and inoculated with fractions containing eBKPyV or naked BKPyV. Infection was assessed by immunofluorescence 2 days postinfection. Results are expressed as percentages of infection and are reported as the means ± standard deviations from six independent experiments.

Several studies have suggested that polysialylated receptors, in particular gangliosides, play an important role in the initial interaction between BKPyV and target cells (9, 10). Since eBKPyVs are surrounded by a lipid bilayer, we hypothesized that they use an alternative entry pathway to infect target cells, compared to naked BKPyV. This hypothesis was supported by electron microscopy experiments which clearly demonstrated not only the docking of membrane-wrapped viral particles at the plasma membrane of target cells (Fig. 2C and D) but also the presence of intact vesicles carrying virions in endosomal compartments (Fig. 3A). It has been shown previously that naked BKPyV agglutinates human type O red blood cells (RBCs) through interactions between the VP1 capsid protein and the gangliosides displayed at the surface of these cells (11). We thus investigated whether eBKPyVs were able to agglutinate human type O RBCs. As expected, we observed that naked BKPyV agglutinated RBCs and that chloroform treatment had no effect on this ability (Fig. 3B). In contrast, viral particles contained in the eBKPyV fraction were able to agglutinate RBCs only after extraction with chloroform, suggesting that they do not use polysialylated gangliosides as an attachment factor. To confirm this result, we treated naive Vero cells with increasing concentrations of neuraminidase prior to incubation with eBKPyV or naked BKPyV. As shown in Fig. 3C, neuraminidase treatment efficiently inhibited naked virion entry in a dose-dependent manner whereas it had only a slight effect on infection with eBKPyV. These findings indicate that the entry of eBKPyV is not dependent on the presence of cell surface sialic acids.

### eBKPyVs are efficiently inhibited by neutralizing antibodies.

**FIG 4:**
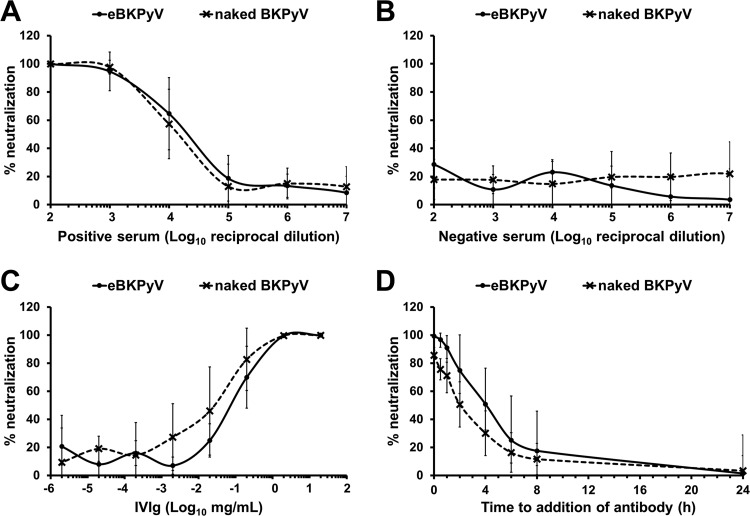
eBKPyVs are efficiently inhibited by neutralizing antibodies. (A) Fractions containing eBKPyV or naked BKPyV were preincubated for 2 h at 37°C with serial dilutions of a BKPyV-seropositive patient serum. Then, mixtures were put into contact with naive cells. Infectivity was assessed 72 h postinfection. Results are expressed as percentages of neutralization and are reported as the means ± standard deviations from 10 independent experiments. (B) A similar experiment was performed using the serum of a 1-year-old individual seronegative for BKPyV. (C) A similar experiment was performed using increasing concentrations of IVIg. Results are expressed as percentages of neutralization and are reported as the means ± standard deviations from two independent experiments. (D) Naked BKPyVs or eBKPyVs were inoculated into naive cells, and IVIg was added at different times postinoculation (final concentration, 2 mg/ml). Infectivity was assessed 48 h postinfection. Results are expressed as percentages of neutralization and are reported as the means ± standard deviations from six independent experiments.

Similarly, we wondered whether the membranes surrounding eBKPyV could render these particles resistant to neutralizing antibodies. Naked BKPyV and eBKPyV were thus preincubated for 2 h with reciprocal dilutions of the serum of a patient seropositive for genotype I of BKPyV and then inoculated into cells for 3 days. As shown in Fig. 4A, under these conditions, we did not find any effect of the membranes surrounding eBKPyVs on the sensitivity to neutralization. The serum of a 1-year-old individual seronegative for BKPyV was used as a negative control, and as expected, no neutralization was observed (Fig. 4B). Furthermore, we also tested the sensitivity to commercially available polyvalent immunoglobulin preparations (IVIg), which are known to contain high titers of potent BKPyV-neutralizing antibodies, and similarly, we did not find any difference between eBKPyVs and naked particles (Fig. 4C). We thus hypothesized that the membrane surrounding eBKPyV must rupture after endocytosis, rendering the capsid accessible to neutralizing antibodies. Consistent with this hypothesis, we observed in a time-of-addition assay that eBKPyVs as well as naked BKPyVs were neutralized when cells were exposed to IVIg either immediately before inoculation with virus or up to 4 h afterward (Fig. 4D). Altogether, these results suggest that naked BKPyVs and eBKPyVs are equally sensitive to neutralizing antibodies which block infection at a postattachment step, after endocytosis.

## DISCUSSION

In this study, we demonstrated that BKPyV uses EVs to be released from infected cells. In recent years, a similar strategy has been described for several viruses that had long been considered nonenveloped, such as hepatitis A and E viruses, coxsackievirus, poliovirus, rotavirus, or norovirus (12–19). The possibility for naked viruses to be released in EVs gives them several advantages: (i) the possibility to be released from infected cells through a nonlytic pathway; (ii) a diversification of the transmission routes, which promotes propagation; (iii) an enhancement of virulence and viral fitness thanks to *en bloc* delivery; and (iv) protection against neutralizing antibodies which target the viral capsid (17–21). Interestingly, it was shown in 1989 that the release of simian virus 40 (SV40) virions from epithelial cells was polarized and occurred without cell lysis (22), and we also observed the release of two populations of SV40 infectious particles, one of which cosedimented with EVs (data not shown). In addition, during the preparation of our manuscript, Morris-Love et al. provided evidence that the JC polyomavirus (JCPyV), which shares 75% sequence homology with BKPyV, also uses EVs as a means of transmission (7). Thus, several members of the polyomavirus family hijack EVs for their release, and the question is raised for other polyomaviruses such as MCPyV and trichodysplasia spinulosa polyomavirus (TSPyV), which are associated with Merkel cell carcinoma and trichodysplasia spinulosa, respectively.

The mechanism leading to the release of eBKPyV has not been deciphered yet. Using the anion channel inhibitor DIDS (4,4′-diisothiocyanatostilbene-2,2′-disulfonate), Evans et al. (6) described that BKPyV is released by a nonlytic pathway, and we think that this may correspond to the release of eBKPyV. Importantly, this mechanism could contribute to the asymptomatic persistence of BKPyV in immunocompetent individuals. We noticed that eBKPyV and naked BKPyV were released by HRPTE cells from 5 days postinfection whereas cell lysis was observed from 15 days postinfection. Furthermore, when testing the effect of DIDS in our model, we observed that it inhibited not only the release of eBKPyV but also that of naked BKPyV (data not shown). Altogether, these results suggest that most naked BKPyVs could come from the disruption of the membranes surrounding eBKPyVs. However, we cannot exclude the chance that the lysis of just a few infected cells could be responsible for the release of naked virions, and in our hands, the toxic concentration of DIDS was too close to the effective concentration to draw firm conclusions. Further studies are thus needed to fully elucidate the mechanism leading to the release of eBKPyV and naked BKPyV. Previous studies have shown that some viruses hijacking EVs use the ESCRT machinery to bud into MVBs (14, 20, 21, 23–25). Since we observed the presence of viral particles in MVBs, it is tempting to speculate on the involvement of this machinery in the release of eBKPyVs. Alternatively, the autophagy process could be involved, as described for other viruses (15, 16). The investigation of the eBKPyV release pathway could enable the identification of new therapeutic targets to prevent BKVN. For instance, it has been proposed that targeting exosome biogenesis and release may have potential clinical implications for cancer therapy (26). The interaction between viral proteins and the ESCRT machinery has also been proposed as a potential target for antiviral therapy to fight against enveloped viruses (27, 28) and also EV-associated naked viruses (29).

Naked BKPyVs are known to use gangliosides for their attachment and entry into target cells (10, 30). In contrast, we clearly showed by electron microscopy that eBKPyVs were able to dock at the plasma membrane and to be endocytosed without interacting with cell surface sialylated glycans. Instead, the lipids of EV membranes (e.g., phosphatidylserine) may play a role, but this remains to be demonstrated (19). Thus, naked virions and eBKPyVs use different entry pathways, which could play a critical role in the dissemination and spread of BKPyV not only during primary infection but also during BKVN. Such an alternative mechanism of infection has also been elegantly demonstrated for EV-associated JCPyV (7). Besides, it has been proposed that this plays a critical role in the dissemination and spread of JCPyV both to and within the central nervous system (7, 31).

For some viruses, *en bloc* delivery enables enhancement of the specific infectivity and viral fitness of viruses thanks to genetic cooperativity among viral quasispecies (17–19). We did not observe such an increased specific infectivity (i.e., infectivity normalized to VP1 content) in our model. However, it is important to note that we used the Dunlop strain, which contains a rearranged noncoding control region and is highly adapted to cell culture. From our point of view, it would be more suitable to investigate a potential enhancement of the viral fitness in the context of an archetypal strain.

Some studies have evaluated the benefit of administering intravenous immunoglobulin preparations containing high titers of potent BKPyV-neutralizing antibodies to patients, in conjunction with reduced immunosuppression (32). However, these clinical studies are difficult to evaluate because of many caveats such as the existence of other concurrent antiviral interventions or widely variable, empirical dosing (32). It has also been suggested that prevaccinating prospective kidney transplant recipients with a multivalent virus-like-particle-based vaccine against all serotypes might offer protection against graft loss or dysfunction due to BKVN (2). We expected that the membranes surrounding eBKPyVs would protect them from neutralization by antibodies. Such results were obtained with EV-associated JCPyV by Morris-Love et al. (7). In contrast, we did not find any difference when we compared the sensitivities of eBKPyV and naked BKPyV to neutralization, by performing dose-response curves with the serum of a seropositive patient or commercially available IVIg preparations. However, we observed that the naked and enveloped BKPyVs were neutralized by the serum up to 4 h after inoculation, suggesting that a postattachment step was blocked by neutralizing antibodies. Thus, it is likely that neutralization occurs after cointernalization and vesicle membrane disruption, as already shown for other viruses, such as hepatitis A virus (HAV) (14, 19). We plan to decipher the mechanisms by which neutralizing antibodies inhibit eBKPyV and naked BKPyV, but an in-depth study will be required to identify the precise entry step that is targeted and to demonstrate that a membrane rupture step occurs for eBKPyV. In any case, our study provides a new view of BKPyV pathogenesis, which raises important questions about the prevention strategies that are based on the induction or administration of neutralizing antibodies. eBKPyV will have to be considered for future studies on BKPyV neutralization.

## MATERIALS AND METHODS

### Cell culture.

Vero (CCL-81) cells were obtained from the American Type Culture Collection and cultured in Dulbecco’s modified Eagle’s medium (DMEM; Invitrogen) supplemented with 10% fetal bovine serum (FBS). HRPTE cells were obtained from Clinisciences (4100-sc) and cultured in renal epithelial cell growth medium (REGM; Lonza). All cells were grown at 37°C in a humidified environment with 5% CO_2_.

### Antibodies and reagents.

The anti-T-antigen (anti-AgT) mouse monoclonal antibody (PAb416) was purchased from Abcam. The 3B2 monoclonal anti-BKPyV VP1 antibody, the anti-mouse IgG (whole-molecule)–peroxidase antibody produced in rabbit, and the neuraminidase were purchased from Sigma. The Alexa Fluor Plus 488-conjugated goat anti-mouse IgG(H+L) was purchased from ThermoFisher. The monoclonal anti-CD63 antibody (MX-49.129.5), the monoclonal anti-calnexin antibody (AF18), and 4′,6-diamidino-2-phenylindole (DAPI) were purchased from Santa Cruz Biotechnology. The monoclonal anti-CD81 antibody (5A6) was kindly provided by J. Dubuisson (Center for Infection and Immunity of Lille, France). The polyclonal anti-CD9 antibody (GTX55564) was purchased from GeneTex. The monoclonal anti-GM130 antibody (35/GM130) was purchased from BD Biosciences.

### BKPyV production.

The plasmid BKV-pUC19 (kindly provided by W. J. Atwood, Brown University, USA) was used to produce the BKPyV. It was obtained from pBKv(34-2) (Dunlop strain, genotype I) as described previously (33). The plasmid was digested with 2 U of BamHI (New England Biolabs) for every 1 μg of DNA for 4 h at 37°C to separate the BKPyV genome from the backbone plasmid. The DNA was then incubated at 65°C to inactivate the enzyme, and it was transfected into Vero cells using Lipofectamine (Invitrogen) as described in the manufacturer’s instructions. Cells were cultured for approximately 4 weeks until a cytopathic effect was observed. Then, BKPyV was amplified by successive infections of naive cells at a multiplicity of infection (MOI) of 1, every 4 days. Extracellular and intracellular viral particles were harvested, extracted by chloroform treatment (34), and filtered at 0.45 μm. The titers of viral stocks were determined by the 50% tissue culture infective dose (TCID_50_) method using immunofluorescence (see below). To produce eBKPyV and naked BKPyV, Vero or HRPTE cells were infected with BKPyV at an MOI of 1. The supernatants were harvested several days postinfection, filtered at 0.45 μm, and overlaid on iodixanol gradients as described below.

### Buoyant density iodixanol gradient ultracentrifugation.

The supernatants of infected cells were overlaid on iodixanol gradients formed by equal-volume (2.3-ml) steps of 20, 25, 30, 35, 40, and 45% (wt/vol) iodixanol (Visipaque, 320 mg/ml; GE Healthcare) solutions in phosphate-buffered saline (PBS). Equilibrium was reached by ultracentrifugation for 24 h at 130,000 × *g* in an SW32.1 Ti rotor at 4°C in a Beckman Optima L-100 K BioSafe ultracentrifuge. Seventeen fractions (1 ml) were collected from the top. The density (g/ml) of each fraction was calculated according to the optical density at 340 nm. AChE activity was measured using Ellman’s method to detect the presence of EVs in each fraction (35). Briefly, 50 μl of each fraction was incubated at 25°C for 15 min with 150 μl of Ellman solution containing 1 mM acetylthiocholine iodide, 0.23 mM 5,5′-dithiobis(2-nitrobenzoic acid), and 0.45 mM NaHCO_3_, all purchased from Sigma. The absorbance was measured at 405 nm. The infectivity of each fraction was assessed as described below.

### Infectivity assays.

Twenty microliters of the fractions recovered after iodixanol gradients was incubated with Vero cells in 96-well plates. At 72 h postinfection, cells were washed with PBS, fixed with paraformaldehyde (3.7% in PBS), and permeabilized with Triton X-100 (0.05% in CSK buffer). Infected cells were detected by immunofluorescence staining of the AgT. Nuclei were stained with DAPI. Immunostained cells were observed with a Zeiss Axio Vert.A1 microscope equipped with Colibri 7 LED illumination. Fluorescent signals were collected with an AxioCam 305 color camera (Zeiss). Percentages of infected cells were automatically determined using the QuantIF ImageJ macro (36).

### Proteinase K protection assay.

Fractions of interest were treated with different concentrations of proteinase K (Qiagen) on ice for 10 min. The reaction was stopped by addition of a 10× solution of protease inhibitor (Pierce protease inhibitor tablets) and by 2× Laemmli buffer (Sigma). The VP1 capsid protein was then detected by Western blotting using the 3B2 monoclonal anti-BKV VP1 antibody (Sigma). The bands were quantified using ImageJ.

### Electron microscopy.

Viral particles contained in the supernatants of infected cells were concentrated 100× using PEG precipitation. Briefly, 300 ml of infected cell supernatant was mixed with 75 ml of a PEG 6000 solution (40% in PBS). The supernatant of naive cells was used as a negative control. The mixtures were incubated overnight at 4°C and centrifuged at 1,500 × *g*, 4°C, for 30 min. The pellets were resuspended in 3 ml of PBS and fractionated by iodixanol gradient as described above. Cells were trypsinized and incubated with the fractions of interest for 2 h at 37°C, under gentle shaking. Then, they were washed with PBS, fixed in 4% paraformaldehyde and 1% glutaraldehyde in 0.1 M phosphate buffer (pH 7.2) for 48 h, and postfixed with 2% osmium tetroxide (Electron Microscopy Sciences, Hatfield, PA, USA) for 1 h. They were then dehydrated in a graded series of ethanol solutions, and cell pellets were embedded in Epon resin, which was allowed to polymerize for 48 h at 60°C. Ultrathin sections were cut on an ultramicrotome (Reichert, Heidelberg, Germany), collected on copper grids, and stained with 5% uranyl acetate-5% lead citrate. The grids were observed with a JEOL JEM-1011 electron microscope (JEOL, Tokyo, Japan) connected to a Gatan Rio 9 digital camera driven by Digital Micrograph software (Gatan, Pleasanton, CA, USA).

### Hemagglutination assays.

RBCs from type O Rh^+^ blood donors were washed three times and suspended in PBS at a final concentration of 0.67% (vol/vol). Then, 50 μl of the suspension was mixed with 25 μl of serial dilutions of eBKPyV or naked BKPyV fractions, previously extracted with chloroform or left untreated. Mixtures were allowed to settle overnight at 4°C, in round-bottom 96-well plates.

### Neutralization assays.

Naked BKPyVs or eBKPyVs were preincubated for 2 h at 37°C with serial dilutions of the serum of a seropositive patient (subtype Ia). Then, the mixtures were put in contact with target cells. Infectivity was assessed 3 days postinfection by immunofluorescence, as described above. The serum of a seronegative 1-year-old individual was used as a negative control. A similar experiment was also performed using increasing concentrations of polyvalent immunoglobulins (Hizentra, 200 mg/ml; CSL Behring).

## References

[1] Chong S, Antoni M, Macdonald A, Reeves M, Harber M, Magee CN 2019 BK virus: current understanding of pathogenicity and clinical disease in transplantation. Rev Med Virol 29:e2044. doi:10.1002/rmv.2044. 30958614

[2] Pastrana DV, Brennan DC, Cuburu N, Storch GA, Viscidi RP, Randhawa PS, Buck CB 2012 Neutralization serotyping of BK polyomavirus infection in kidney transplant recipients. PLoS Pathog 8:e1002650. doi:10.1371/journal.ppat.1002650. 22511874 PMC3325208

[3] Solis M, Velay A, Porcher R, Domingo-Calap P, Soulier E, Joly M, Meddeb M, Kack-Kack W, Moulin B, Bahram S, Stoll-Keller F, Barth H, Caillard S, Fafi-Kremer S 2018 Neutralizing antibody-mediated response and risk of BK virus-associated nephropathy. J Am Soc Nephrol 29:326–334. doi:10.1681/ASN.2017050532. 29042457 PMC5748919

[4] Helle F, Brochot E, Handala L, Martin E, Castelain S, Francois C, Duverlie G 2017 Biology of the BKPyV: an update. Viruses 9:e327. doi:10.3390/v9110327. 29099746 PMC5707534

[5] Bennett SM, Zhao L, Bosard C, Imperiale MJ 2015 Role of a nuclear localization signal on the minor capsid proteins VP2 and VP3 in BKPyV nuclear entry. Virology 474:110–116. doi:10.1016/j.virol.2014.10.013. 25463609 PMC4259852

[6] Evans GL, Caller LG, Foster V, Crump CM 2015 Anion homeostasis is important for non-lytic release of BK polyomavirus from infected cells. Open Biol 5:e150041. doi:10.1098/rsob.150041. PMC4554916 26246492

[7] Morris-Love J, Gee GV, O’Hara BA, Assetta B, Atkinson AL, Dugan AS, Haley SA, Atwood WJ 2019 JC polyomavirus uses extracellular vesicles to infect target cells. mBio 10:e00379-19. doi:10.1128/mBio.00379-19. 30967463 PMC6456752

[8] Feng HC, Kwun HJ, Liu X, Gjoerup O, Stolz DB, Chang Y, Moore PS 2011 Cellular and viral factors regulating Merkel cell polyomavirus replication. PLoS One 6:e22468. doi:10.1371/journal.pone.0022468. 21799863 PMC3142164

[9] Low JA, Magnuson B, Tsai B, Imperiale MJ 2006 Identification of gangliosides GD1b and GT1b as receptors for BK virus. J Virol 80:1361–1366. doi:10.1128/JVI.80.3.1361-1366.2006. 16415013 PMC1346969

[10] Neu U, Allen SA, Blaum BS, Liu Y, Frank M, Palma AS, Stroh LJ, Feizi T, Peters T, Atwood WJ, Stehle T 2013 A structure-guided mutation in the major capsid protein retargets BK polyomavirus. PLoS Pathog 9:e1003688. doi:10.1371/journal.ppat.1003688. 24130487 PMC3795024

[11] Sinibaldi L, Viti D, Goldoni P, Cavallo G, Caroni C, Orsi N 1987 Inhibition of BK virus haemagglutination by gangliosides. J Gen Virol 68:879–883. doi:10.1099/0022-1317-68-3-879. 3029311

[12] Takahashi M, Tanaka T, Takahashi H, Hoshino Y, Nagashima S, Jirintai Mizuo H, Yazaki Y, Takagi T, Azuma M, Kusano E, Isoda N, Sugano K, Okamoto H 2010 Hepatitis E virus (HEV) strains in serum samples can replicate efficiently in cultured cells despite the coexistence of HEV antibodies: characterization of HEV virions in blood circulation. J Clin Microbiol 48:1112–1125. doi:10.1128/JCM.02002-09. 20107086 PMC2849599

[13] Bird SW, Maynard ND, Covert MW, Kirkegaard K 2014 Nonlytic viral spread enhanced by autophagy components. Proc Natl Acad Sci U S A 111:13081–13086. doi:10.1073/pnas.1401437111. 25157142 PMC4246951

[14] Feng Z, Hensley L, McKnight KL, Hu F, Madden V, Ping L, Jeong SH, Walker C, Lanford RE, Lemon SM 2013 A pathogenic picornavirus acquires an envelope by hijacking cellular membranes. Nature 496:367–371. doi:10.1038/nature12029. 23542590 PMC3631468

[15] Robinson SM, Tsueng G, Sin J, Mangale V, Rahawi S, McIntyre LL, Williams W, Kha N, Cruz C, Hancock BM, Nguyen DP, Sayen MR, Hilton BJ, Doran KS, Segall AM, Wolkowicz R, Cornell CT, Whitton JL, Gottlieb RA, Feuer R 2014 Coxsackievirus B exits the host cell in shed microvesicles displaying autophagosomal markers. PLoS Pathog 10:e1004045. doi:10.1371/journal.ppat.1004045. 24722773 PMC3983045

[16] Chen YH, Du W, Hagemeijer MC, Takvorian PM, Pau C, Cali A, Brantner CA, Stempinski ES, Connelly PS, Ma HC, Jiang P, Wimmer E, Altan-Bonnet G, Altan-Bonnet N 2015 Phosphatidylserine vesicles enable efficient en bloc transmission of enteroviruses. Cell 160:619–630. doi:10.1016/j.cell.2015.01.032. 25679758 PMC6704014

[17] Altan-Bonnet N 2016 Extracellular vesicles are the Trojan horses of viral infection. Curr Opin Microbiol 32:77–81. doi:10.1016/j.mib.2016.05.004. 27232382 PMC4983493

[18] Santiana M, Ghosh S, Ho BA, Rajasekaran V, Du WL, Mutsafi Y, De Jesus-Diaz DA, Sosnovtsev SV, Levenson EA, Parra GI, Takvorian PM, Cali A, Bleck C, Vlasova AN, Saif LJ, Patton JT, Lopalco P, Corcelli A, Green KY, Altan-Bonnet N 2018 Vesicle-cloaked virus clusters are optimal units for inter-organismal viral transmission. Cell Host Microbe 24:208–220. doi:10.1016/j.chom.2018.07.006. 30092198 PMC6226266

[19] Altan-Bonnet N, Perales C, Domingo E 2019 Extracellular vesicles: vehicles of en bloc viral transmission. Virus Res 265:143–149. doi:10.1016/j.virusres.2019.03.023. 30928427

[20] Feng Z, Hirai-Yuki A, McKnight KL, Lemon SM 2014 Naked viruses that aren’t always naked: quasi-enveloped agents of acute hepatitis. Annu Rev Virol 1:539–560. doi:10.1146/annurev-virology-031413-085359. 26958733 PMC12175490

[21] Feng Z, Lemon SM 2014 Peek-a-boo: membrane hijacking and the pathogenesis of viral hepatitis. Trends Microbiol 22:59–64. doi:10.1016/j.tim.2013.10.005. 24268716 PMC3918648

[22] Clayson ET, Brando LV, Compans RW 1989 Release of simian virus 40 virions from epithelial cells is polarized and occurs without cell lysis. J Virol 63:2278–2288. 2539518 10.1128/jvi.63.5.2278-2288.1989 PMC250646

[23] Okamoto H 2013 Culture systems for hepatitis E virus. J Gastroenterol 48:147–158. doi:10.1007/s00535-012-0682-0. 23104469 PMC3698424

[24] Holla RP, Ahmad I, Ahmad Z, Jameel S 2013 Molecular virology of hepatitis E virus. Semin Liver Dis 33:3–14. doi:10.1055/s-0033-1338110. 23564385

[25] Nagashima S, Jirintai S, Takahashi M, Kobayashi T, Tanggis, Nishizawa T, Kouki T, Yashiro T, Okamoto H 2014 Hepatitis E virus egress depends on the exosomal pathway, with secretory exosomes derived from multivesicular bodies. J Gen Virol 95:2166–2175. doi:10.1099/vir.0.066910-0. 24970738

[26] Datta A, Kim H, McGee L, Johnson AE, Talwar S, Marugan J, Southall N, Hu X, Lal M, Mondal D, Ferrer M, Abdel-Mageed AB 2018 High-throughput screening identified selective inhibitors of exosome biogenesis and secretion: a drug repurposing strategy for advanced cancer. Sci Rep 8:8161. doi:10.1038/s41598-018-26411-7. 29802284 PMC5970137

[27] Siarot L, Chutiwitoonchai N, Sato H, Chang H, Sato H, Fujino M, Murakami T, Aono T, Kodama E, Kuroda K, Takei M, Aida Y 2018 Identification of human immunodeficiency virus type-1 Gag-TSG101 interaction inhibitors by high-throughput screening. Biochem Biophys Res Commun 503:2970–2976. doi:10.1016/j.bbrc.2018.08.079. 30126636

[28] Tavassoli A, Lu Q, Gam J, Pan H, Benkovic SJ, Cohen SN 2008 Inhibition of HIV budding by a genetically selected cyclic peptide targeting the Gag-TSG101 interaction. ACS Chem Biol 3:757–764. doi:10.1021/cb800193n. 19053244

[29] Anang S, Kaushik N, Hingane S, Kumari A, Gupta J, Asthana S, Shalimar, Nayak B, Ranjith-Kumar CT, Surjit M 2018 Potent inhibition of hepatitis E virus release by a cyclic peptide inhibitor of the interaction between viral open reading frame 3 protein and host tumor susceptibility gene 101. J Virol 92:e00684-18. doi:10.1128/JVI.00684-18. 30068652 PMC6158408

[30] Low J, Humes HD, Szczypka M, Imperiale M 2004 BKV and SV40 infection of human kidney tubular epithelial cells in vitro. Virology 323:182–188. doi:10.1016/j.virol.2004.03.027. 15193914

[31] Santiana M, Altan-Bonnet N 2019 Insane in the membrane: glial extracellular vesicles transmit polyomaviruses. mBio 10:e01024-19. 31138755 10.1128/mBio.01024-19 PMC6538792

[32] Hirsch HH, Randhawa PS 2019 BK polyomavirus in solid organ transplantation—guidelines from the American Society of Transplantation Infectious Diseases Community of Practice. Clin Transplant 33:e13528. doi:10.1111/ctr.13528. 30859620

[33] Dugan AS, Gasparovic ML, Tsomaia N, Mierke DF, O’Hara BA, Manley K, Atwood WJ 2007 Identification of amino acid residues in BK virus VP1 that are critical for viability and growth. J Virol 81:11798–11808. doi:10.1128/JVI.01316-07. 17699578 PMC2168807

[34] Deutsch DR, Wassermann FE 1965 Modification of the procedure to determine the sensitivity of viruses to chloroform. Appl Microbiol 13:1040–1041. doi:10.1128/AEM.13.6.1040-1041.1965. 4286246 PMC1058393

[35] Ellman GL, Courtney KD, Andres V Jr, Feather-Stone RM 1961 A new and rapid colorimetric determination of acetylcholinesterase activity. Biochem Pharmacol 7:88–95. doi:10.1016/0006-2952(61)90145-9. 13726518

[36] Handala L, Fiore T, Rouillé Y, Helle F 2019 QuantIF: an ImageJ macro to automatically determine the percentage of infected cells after immunofluorescence. Viruses 11:e165. doi:10.3390/v11020165. 30791409 PMC6410121

